# Isoacids supplementation improves growth performance and feed fiber digestibility associated with ruminal bacterial community in yaks

**DOI:** 10.3389/fmicb.2023.1175880

**Published:** 2023-06-15

**Authors:** Fei Jiang, Yanhua Gao, Zhongli Peng, Xiulian Ma, Yinjie You, Zhibin Hu, Anxiang He, Yupeng Liao

**Affiliations:** ^1^College of Animal and Veterinary Sciences, Southwest Minzu University, Chengdu, China; ^2^Key Laboratory of Animal Science of National Ethnic Affairs Commission, Chengdu, China; ^3^Key Laboratory of Qinghai-Tibetan Plateau Animal Genetic Resources Reservation and Utilization, Chengdu, China; ^4^Qinghai-Tibetan Plateau Animal Genetic Resource Reservation and Utilization Key Laboratory of Sichuan Province, Chengdu, China; ^5^Institute of Animal Husbandry Science, Ganzi Tibetan Autonomous Prefecture, Kangding, China; ^6^Si Chuan Action Biotech Co., Ltd., Guanghan, China

**Keywords:** yak (*Bos grunniens*), isoacids, growth performance, fiber digestibility, ruminal bacterial community, 16S rRNA gene sequencing

## Abstract

**Introduction:**

This study was conducted to assess the effect of mixed isoacid (MI) supplementation on fermentation characteristics, nutrient apparent digestibility, growth performance, and rumen bacterial community in yaks.

**Methods:**

A 72-h *in vitro* fermentation experiment was performed on an ANKOM RF gas production system. MI was added to five treatments at doses of 0, 0.1%, 0.2%, 0.3%, 0.4%, and 0.5% on the dry matter (DM) basis of substrates using a total of 26 bottles (4 bottles per treatment and 2 bottles as the blank). Cumulative gas production was measured at 4, 8, 16, 24, 36, 48, and 72 h. Fermentation characteristics including pH, the concentration of volatile fatty acids (VFAs), ammonia nitrogen (NH_3_-N), microbial proteins (MCP), and the disappearance rate of dry matter (DMD), neutral detergent fiber (NDFD), and acid detergent fiber (ADFD) were measured after a 72-h *in vitro* fermentation to determine an optimal MI dose. Fourteen Maiwa male yaks (180–220 kg, 3–4 years old of age) were randomly assigned to the control group (without MI, *n* = 7) and the supplemented MI group (*n* = 7, supplemented with 0.3% MI on DM basis) for the 85-d animal experiment. Growth performance, nutrient apparent digestibility, rumen fermentation parameters, and rumen bacterial diversity were measured.

**Results:**

Supplementation with 0.3% MI achieved the greatest propionate and butyrate content, NDFD and ADFD compared with other groups (*P* < 0.05). Therefore, 0.3% was used for the animal experiment. Supplementation with 0.3% MI significantly increased the apparent digestibility of NDF and ADF (*P* < 0.05), and the average daily weight gain of yaks (*P* < 0.05) without affecting the ruminal concentration of NH_3_-N, MCP, and VFAs. 0.3% MI induced rumen bacteria to form significantly different communities when compared to the control group (*P* < 0.05). g__norank_f__*Bacteroidales*_BS11_gut_group, g__norank_f__*Muribaculaceae*, g__*Veillonellaceae*_UCG-001, g__*Ruminococcus*_*gauvreauii*_group, g__norank_f__norank_o__RF39 and g__*Flexilinea* were identified as the biomarker taxa in responding to supplementation with 0.3% MI. Meanwhile, the abundance of g__*Flexilinea* and g__norank_f__norank_o__RF39 were significantly positively correlated with the NDF digestibility (*P* < 0.05).

**Conclusion:**

In conclusion, supplementation with 0.3% MI improved the *in vitro* rumen fermentation characteristics, feed fiber digestibility, and growth performance in yaks, which was associated with changes of the abundance of g__*Flexilinea* and g__norank_f__norank_o__RF39.

## 1. Introduction

Yaks (*Bos grunnien*s), a large ruminant, have adapted well to the high-altitude, low oxygen, and cold environment of the Qinghai-Tibet Plateau, and provide milk, meat, wool, leather, and labor for local residents (Jing et al., 2022). The adaptation of yaks to the highlands is closely related to their unique characteristics in terms of digestion, physiology, rumen fermentation, and rumen microbiota composition. It has been found that yaks have a larger heart and lung volume, a higher capacity to sense the environment, and a higher energy metabolism than cattle (Wang et al., 2011). In addition, yaks are more tolerant to roughage than cattle (Shao et al., 2010), e.g., they digest neutral detergent fiber (NDF) better than cattle and they use nitrogen more efficiently (Zhou et al., 2017, 2018). Yaks are mainly reared on natural pastures, but due to the harsh climatic conditions in the winter season, this feeding pattern cannot sustain adequate nutrient and energy intake for yaks throughout the year, resulting in a retarded growth rate and even weight loss (Long et al., 2005; Ding et al., 2012). Therefore, barn- or feedlot-feeding was introduced to conserve natural pastures, improve the growth rate, and shorten the feeding period of yaks (Kang et al., 2020; Du et al., 2021; Hu et al., 2021).

Similar to other ruminants, yaks rely on rumen microorganisms to break down dietary nutrient components. Differences in physiological characteristics and digestibility suggest that the rumen microbiome composition of yaks may differ from that of other ruminants (Xin et al., 2019). Compared with cattle, yaks show a higher abundance of *Firmicutes* in the rumen (Xue et al., 2018). A representative genus is *Clostridium*, which can degrade cellulose, xylan or pectin, and other substances in the rumen (Cholewińska et al., 2020). The ruminal bacterial community of yaks also showed better dietary fiber degradation features compared with the normal cattle or hybrid cattle-yak (Dai et al., 2021; Liu et al., 2022). This may contribute to improving the rumen fermentation characteristics of yaks, for example, yaks showed greater ruminal production of VFA but achieved lower methane emission when compared with cattle and sheep (Zhang et al., 2016). Therefore, altering the composition and structure of the bacterial community may improve the rumen fermentation characteristics, which is beneficial for the growth performance of yaks.

Isoacids, a term referring to the branched-chain volatile fatty acids (BCVFAs), namely, isovaleric acid (isovalerate), isobutyric acid (isobutyrate), and 2-methylbutyric acid (2-methybutyrate), and the straight-chain valeric acid (valerate), are the microbial metabolites of amino acids, including valine, leucine, isoleucine, and proline (Andries et al., 1987; Apajalahti et al., 2019). Previous studies have shown that the addition of isoacids to ruminant diets, alone or in combination, favors increased enzymatic activities and proliferation of ruminal fiber-degrading bacteria, and promotes crude fiber digestibility *in vivo* (Liu et al., 2014, 2020; Wang et al., 2015, 2018a; Liu C. S. et al., 2016). Furthermore, in an *in vitro* continuous fermentation model, supplementation of a mixture of BCVFAs showed improved NDF digestibility compared with the addition of individual isoacids (Roman-Garcia et al., 2021a,b) and stimulated the relative abundance of fiber-degrading bacteria, such as *Fibrobacter* and *Treponema* (Roman-Garcia et al., 2021c). Given these beneficial effects of isoacids on rumen fiber degradation, we hypothesize that supplementation with isoacids could be an effective option to promote feed fiber utilization in barn-feeding yaks. Therefore, the objective of this study was to evaluate the effects of supplementing a mixture of isoacids on growth performance, rumen fermentation characteristics, and nutrient digestibility in yaks. Considering the metabolic process of isoacids by microorganisms in the rumen, we evaluated the changes in the ruminal bacterial community in response to isoacid supplementation using 16s rRNA gene sequencing and correlated the ruminal microbiome with fermentation metabolites and fiber digestibility in yaks.

## 2. Material and methods

### 2.1. Mixed isoacids supplement

The supplemental mixture of isoacids used in this study is a commercially available feed additive product (Yangleyou, provided by Si Chuan Action Biotech Co., Ltd), which consisted of a mixture of isobutyrate, isovalerate, valerate, and 2-methylbutyrate. The effective content of mixed isoacid (MI) was 63.6 g/100 g. The liquid mixture of isoacids was absorbed using silica as the carrier in a ratio of 64:36.

### 2.2. *In vitro* fermentation experiment and cumulative gas production measurement

An *in vitro* fermentation experiment was conducted to evaluate the role of different supplemental levels of MI on the fiber disappearance rate and the fermentation characteristics. The treatment groups included a non-supplementation control group (CON) and five MI-supplemented groups supplemented with MI on the dry matter (DM) basis of the substrate, namely: 0.1% MI (treatment group 1, T1), 0.2% MI (treatment group 2, T2), 0.3% MI (treatment group 3, T3), 0.4% MI (treatment group 4, T4), and 0.5% MI (treatment group 5, T5). Each treatment group contained four replicates.

The *in vitro* fermentation experiment was performed using the ANKOM RF Gas Production Systems (ANKOM), which is designed to automatically measure the kinetics of a microbial fermentation by monitoring the gas pressure within multiple modules equipped with temperature sensors and remotely recording the data in computer spreadsheets. The modules can communicate information to a computer using radio frequency (RF) transmission. Numerous variables can be operated from the computer interface such as data recording intervals and the automatic pressure release through internal valves in each module. A run was performed with a total of 24 bottles assigned to 6 groups and 2 bottles as blank. A total of 30 ml of rumen fluid, 120 ml of artificial saliva, and 1.00 g of substrate were added to a glass fermentation bottle and covered with the ANKOM module. A zero-reference module was used to measure ambient pressure. Two additional bottles incubated with rumen fluid and artificial saliva only (no substrate) were used as a blank control to correct for gas production. Briefly, fresh rumen fluid was collected from four male Maiwa yaks through a stainless-steel stomach tube attached to a rumen vacuum sampler as previously reported (Wang et al., 2016a). Yaks were fed the same regime as described in Table 1. Feed particles were discarded by filtering the liquid collection through four layers of sterile gauze. The filtered solutions were then transferred to a vacuum thermobottle, which was pre-incubated at 37°C. Carbon dioxide (CO_2_) was continuously injected into the rumen fluid to maintain an anaerobic atmosphere during all of the processes. Artificial saliva solution was prepared according to the previous procedure (Menke et al., 1979) and well mixed with rumen fluid in a ratio of 1:4 while simultaneously streaming with CO_2_. The fermentation bottles and modules were stabilized at 39°C for 1 h, then added with substrates and 150 ml of the mixing liquids, and incubated at 39°C, 110 rpm/min for 72 h. Real-time absolute gas pressure and temperature of each module were recorded every 15 min during the 72 h of fermentation. The cumulative gas production at 4, 8, 16, 24, 48, and 72 h was converted according to the ANKOM manual using the following equation:


$$\begin{array}{l} {\text{V}}_{\text{x}}{=\text{V}}_{\text{j}}\;\times \;{\text{P}}_{\text{psi}}\;\times \;0.\text{068 }004\;084 \end{array}$$

**Table 1:** Ingredients and chemical composition of the TMR^1^.

**Items**	**Content**
**Ingredient composition, g/100 g DM**	
Corn	31.00
Soybean meal	13.00
Urea	0.15
Na_2_SO_4_	0.35
NaCl	0.35
Premix^2^	1.90
Rumen-protected lysine	0.15
Rumen-protected methionine	0.10
Yeast culture	0.50
Small peptide	0.50
Hydrogenated palm oil	2.00
Corn silage	50.00
Total	100.00
**Nutrient composition, g/100 g DM** ^3^	
DM	95.10
EE	3.15
CP	14.31
NDF	30.13
ADF	12.16
Ga	0.76
P	0.38

TMR, Total mixed ration; DM, dry matter; NDF, neutral detergent fiber; ADF, acid detergent fiber; CP, crude protein; EE, ether extract; Ca, calcium; P, phosphorus.

The premix (vitamins and microelements) contained the following ingredients as per kg of DM: vitamin A 2 500 IU, vitamin D 550 IU, vitamin E 10 IU, copper 10 mg, iron 50 mg, manganese 40 mg, zinc 40 mg, iodine 0.5 mg, selenium 0.2 mg, and cobalt 0.2 mg.

The nutrient composition is based on all measured values.

^1^An explanation of abbreviations for Total mixed ration. ^2^An explanation of the ingredients content of premix in per kg feed at dry matter basis. ^3^An explanation of abbreviations for dry matter.

where *V*_x_ = gas production volume, *V*_j_ = volume of space above the liquid surface inside the bottle, and P_psi_ = gas pressure, recorded by the ANKOM GPM software (1 psi≈ 6.89 kPa).

### 2.3. *In vitro* rumen fermentation characteristics and nutrient disappearance rate

The *in vitro* fermentation experiment was terminated in an ice-water bath after 72 h. The pH value of each fermentation fluid was immediately measured using a pH meter. The fermentation fluid was collected to determine the rumen fermentation characteristics, including the concentration of NH_3_-N, MCP, and VFAs. Briefly, the fermentation fluid was centrifuged at 3,000 rpm for 10 min, and an aliquot of the supernatant was used to determine the concentration of NH_3_-N using the micro-Kjeldahl method (AOAC, 2001). Another aliquot of the supernatant was transferred to a 10-ml centrifuge tube, ultrasonicated (ultrasonic probe = 2 mm, 350 W, repeated 3 times, 30 s each time, and interval = 30 s), and centrifuged at 3,000 rpm at 4°C for 10 min. Briefly, 1 ml of the supernatant was centrifuged at 10,000 rpm at 4°C for 20 min. The precipitate was rinsed twice with 1 ml of saline and then re-suspended with 1 ml of distilled water to determine the concentration of MCP according to the manufacturer's instructions of the BCA kits (Beijing Solarbio Science & Technology Co. Ltd, Beijing, China).

The concentration of VFAs in the fermentation fluid was measured according to the method described by Wang et al. (2014). Briefly, 1.0 ml of the supernatant was acidified with 200 μL metaphosphoric acid (25%) overnight at 4°C. The samples were analyzed on a gas chromatograph (7890B, Agilent Technology Inc., Santa Clara, CA) with a chromatographic column (HP INNOwax - 19091N, 30 m long, 0.32 mm ID, 0.50 m film) according to the manufacturer's protocol. The VFAs content was determined by comparison with the linear retention times of known standards (Shanghai Anpel Experimental Technology Co., LTD, Shanghai, China).

DM, NDF, and acid detergent fiber (ADF) degradability (DMD, NDFD, and ADFD) of the fermentation residue were measured after 72 h of incubation. The DM content was determined according to the AOAC method (AOAC, 2001), and the contents of NDF and ADF were analyzed using a fiber analyzer (automatic fiber determination analyzer, Gernardt F12) as described by Van Soest et al. (1991). Finally, they were calculated using the following equations:


$$\begin{array}{l} \text{DMD }=\;\frac{(\text{DM of substrate }-\text{ DM of residue})\;\times \text{ 100}}{\text{DM of substrate}}\\ \text{NDFD }=\;\frac{(\text{NDF of substrate }-\text{ NDF of residue})\;\times \;100}{\text{NDF of substrate}}\\ \text{ADFD }=\;\frac{(\text{ADF of substrate }-\text{ ADF of residue})\;\times \;100}{\text{ADF of substrate}} \end{array}$$

### 2.4. Animals, management, and sample collection

All animal experimental procedures were approved by the Animal Care and Use Committee of Southwest Minzu University (approval number P20210510-2). The animal experiment was conducted at Seda Niuduoduo Yak Breeding Co. LTD (Ganzi, China, 30°055′ N and 101°969′ E). A total of fourteen Maiwa male yaks (body weight of 180–220 kg and 3–4 years of age) were randomly divided into two groups (*n* = 7 per group), tied and fed individually, and provided with a basal diet (control group; CON) or the basal diet supplemented with 0.3% MI. The Total Mixed Ration (TMR) diet was formulated according to the Feeding Standards of Beef Cattle of China (NY/T 815-2004, Ministry of Agriculture of the People's Republic of China, 2004). The ingredients and nutrient compositions are listed in Table 1. During the 95-day experimental period, the yaks had free access to water and were offered diets *ad libitum* twice a day (at 08:00–09:00 and 17:30–18:00). After 10-days of acclimatization, average dry matter intake (ADMI) was recorded before morning feeding. Animal body weight (BW) was recorded at the beginning and the end of the experiment to calculate the average daily gain (ADG) and feed conversion ratio (FCR).

Feed samples were collected once every 2 weeks and stored at −20°C for nutritional evaluation. Total fecal samples were collected three times daily and pooled on the last 3 days of the experiment. In total, 200 g of total fecal sample was collected from each yak, sampled, and stored at −20°C until analysis. Briefly, the contents of DM, crude protein (CP), acid insoluble ash (AIA), and ether extract (EE), in the diet and fecal samples, were analyzed according to the standard methods described by AOAC (2001). Calcium (Ca) content was measured by EDTA complexometric titration, and total phosphorus (P) content was determined using a spectrophotometer (the People's Republic of China National Standard, 2002a,b). NDF and ADF were analyzed as described in 2.3. AIA was used as an internal marker to evaluate the apparent digestibility of nutrients, which was calculated using the following equation:


$$\begin{array}{l} \text{Nutrient apparent digestibility}=100\times \left(1-\frac{R_{AIA}}{M_{AIA}}\times \frac{M_{n}}{R_{n}}\right) \end{array}$$

where R*n*= nutrient content in the feed, M*n*= nutrient content in the feces, R_AIA_= hydrochloric acid insoluble ash content in the feed, and M_AIA_=hydrochloric acid insoluble ash content in the feces (Cheng et al., 2014).

Rumen fluid was collected through a stainless-steel stomach tube at the end of the experiment after overnight fasting. The first two tubes of ruminal fluid were discarded to avoid saliva contamination. The rumen fluid was then divided into two aliquots. One aliquot (5 ml) was transferred to a cryogenic vial and immediately frozen in liquid nitrogen for 16S rRNA sequencing. The other aliquot (30 ml) was transferred to a centrifuge tube and stored at −80°C for chemical determination of VFAs, NH_3_-N, and MCP concentrations using the same methods described in Section 2.2. Blood samples were collected from the jugular vein of yaks and anticoagulated with heparin sodium. Plasma was collected after centrifugation at 4,000 rpm for 10 min and stored at −80°C for further analysis of biochemical and hormonal parameters.

### 2.5. Rumen fluid microbial DNA extraction, 16S rRNA gene amplification, and bioinformatics analysis

Total microbial genomic DNA from the rumen fluid was extracted using a FastDNA^®^ Spin Kit for soil (MP Biomedicals, USA), according to the standard operating procedures. The concentration, quality, and completeness of the extracted DNA were determined using a spectrophotometer (NanoDrop2000, Thermo Fisher, USA) and 1.0% agarose gel electrophoresis (Biowest Agarose, Biowest, Spain), and then the DNA was stored at −80°C for further use. The hypervariable region V3–V4 of the bacterial 16S rRNA gene was amplified with primer pairs 338F (5′ ACTCCTACGGGAGGCAGCAG-3′) and 806R (5′-GGACTACHVGGGTWTCTAAT-3′) using an ABI GeneAmp^®^ 9700 PCR thermocycler (ABI, CA, USA; Liu Q. et al., 2016). A 20-μL reaction system contained 4 μL of 5 × Fast Pfu buffer, 2 μL of 2.5 mM dNTPs, 0.8 μL of each primer (5 μM), 0.4 μL of Fast Pfu polymerase, 10 ng of template DNA, and ddH_2_O to a final volume of 20 μL. The temperature program was performed as follows: 95°C for 3 min, followed by 27 cycles of denaturation at 95°C for 30 s, 55°C for 30 s, and 72°C for 45 s, a single extension at 72°C for 10 min, and ending at 4°C. Purification and quantification of the amplification product were performed using the AxyPrep DNA Gel Extraction Kit (Axygen Biosciences, Axygen, USA) and the Quantus™ Fluorometer (Promega, USA).

The amplicons were sequenced on an Illumina MiSeq PE300 platform (Illumina, San Diego, CA, USA) at Majorbio (Shanghai, China). Raw sequencing reads were de-multiplexed using an in-house Perl script, quality-filtered using FASTP (v 0.19.6; Chen et al., 2018), and merged using FLASH (v1.2.11; Magoc and Salzberg, 2011). Although chloroplast DNA was not removed during the quality control process, this had a negligible effect on the analysis results. Paired-end reads were merged into a sequence with a minimum overlap length of 10 bp and a maximum mismatch ratio of 0.2 according to the overlapping relationships. The optimized sequences were clustered into operational taxonomic units (OTUs) based on a similarity >97% by using UPARSE 11 (Stackebrandt and Goebel, 1994; Edgar, 2013). To minimize the effect of sequencing depth on the bacterial diversity measure, the number of 16S rRNA gene sequences from each sample was rarefied to 32095, which still resulted in an average Good's coverage of 99.06 ± 0.044%.

The data were analyzed on the online platform of Majorbio Cloud Platform (www.majorbio.com) according to the description found in the study by Ren et al. (2022). The Mothur software programs (version v.1.30.2; Schloss et al., 2009) were applied to calculate the alpha diversity of the bacterial community, including the Sobs, Shannon, Simpson, and Chao indices. Principal coordinates analysis (PCoA) was used to determine the similarity among the microbial communities in different samples based on the Bray-Curtis distance metric using the Vegan v2.5-3 package of R (version 3.3.1). The bacterial community composition and sample-to-taxa relationship were visualized using Circos-0.67-7 (http://circos.ca/). The most discriminative abundant bacterial taxa (from phyla to genera) between groups were identified using a linear discriminant analysis (LDA) effect size (LEfSe; LDA score>3, *P* < 0.05; Segata et al., 2011). The association between the biomarker taxa and the phenotypic parameters was analyzed using Spearman's correlation and visualized using the Pheatmap package of R (version 3.3.1).

### 2.6. Statistical analysis

Data were analyzed using the SPSS (Statistical software package, version 20.0, IBM, USA). A two-way ANOVA used a general linear model (GLM) to examine the effect of treatment on *in vitro* gas production. Duncan's method was used for multiple comparisons. A one-way ANOVA was used to analyze other parameters after data normality was tested using the Shapiro-Wilk test, and polynomial contrasts were generated using a curve estimation regression analysis. The data from the animal experiment were analyzed via the student's *t*-test. The student's *t-*test was used to determine the differential bacterial taxa. Significance was declared at *P* ≤ 0.05, and a significant trend was considered when 0.05 < *P* ≤ 0.10.

### 2.7. Data availability

The raw sequencing reads of the bacterial 16S rRNA amplicon for all of the ruminal fluid samples are deposited in the genome sequence archive (GSA) database under the BioProject accession number PRJCA016021.

## 3. Results

### 3.1. Supplementation with mixed isoacids promoted feed fiber disappearance rate and VFAs production *in vitro*

The addition of different levels of MI did not affect the cumulative *in vitro* gas production volumes, and no interaction between incubation time and treatment was observed (Supplementary Figure 1). Nevertheless, the inclusion of MI strongly affected the NDFD and ADFD (*P* = 0.002 and *P* = 0.004; Table 2). With increasing supplementation, the NDFD and ADFD showed significant quadratic trends (*P* < 0.01). Compared with the control and other supplementation levels, the supplementation with 0.3% MI achieved the greatest NDFD and ADFD (*P* < 0.05).

**Table 2:** Effects of mixed isoacids supplementation on DMD, NDFD, and ADFD of the *in vitro* fermentation experiment.

**Items^1^**	**Groups**						**SEM**	***P*** **-value** ^**2**^		
	**CON**	**T1**	**T2**	**T3**	**T4**	**T5**		**Treatment**	**Linear**	**Quadratic**
DMD	79.77	83.33	83.82	80.88	79.19	79.05	1.763	0.277	0.233	0.129
NDFD	53.14^bc^	58.51^bc^	62.33^ab^	71.49^a^	52.54^bc^	52.35^bc^	3.037	0.002	0.741	0.007
ADFD	31.65^c^	37.26^bc^	44.01^b^	53.88^a^	41.66^bc^	41.49^bc^	3.219	0.004	0.051	0.002

DMD, dry matter degradability; NDFD, neutral detergent fiber degradability; ADFD, acid detergent fiber degradability.

CON: control group (no mixed isoacids added); T1: treatment 1 (0.1% mixed isoacids added); T2: treatment 2 (0.2% mixed isoacids added); T3: treatment 3 (0.3% mixed isoacids added); T4: treatment 4 (0.4% mixed isoacids added); and T5: treatment 5 (0.5% mixed isoacids added).

Different superscripts indicated significant differences within a row (*P* < 0.05).

^1^An explanation of DMD, NDFD and ADFD which were showed in the table. ^2^An explanation that about *P*-value for significant differences.

The effects of supplementing MI on the *in vitro* fermentation characteristics are presented in Table 3. The pH value of the fermentation fluid was strongly affected by MI (*P* < 0.001). When the addition levels were higher than 0.3%, the pH value of the fermentation fluid was significantly lower than that of the control group (*P* < 0.05). The addition of MI showed a quadratic effect on the concentration of NH_3_-N (*P* = 0.021), but not on the concentration of MCP (*P* = 0.685). Propionate and butyrate production displayed a clear quadratic effect in response to MI supplementation (*P* < 0.05). Supplementation with 0.3% MI achieved the highest levels of these two volatile metabolites compared with the control and other supplementation groups (*P* < 0.05). Moreover, the concentrations of total VFAs (TVFA) tended to show a quadratic increase tendency (*P* = 0.055). No marked differences were detected for the production of acetate, valerate, and the ratio of acetate to propionate among the groups (*P* > 0.05). No significant difference was observed in the molar percentage of any of these VFAs (*P* > 0.05).

**Table 3:** Effects of mixed isoacids supplementation on ruminal fermentation characteristics of the *in vitro* fermentation experiment.

**Items^1^**	**Groups** ^**2**^						**SEM**	***P*** **-value** ^**3**^		
	**CON**	**T1**	**T2**	**T3**	**T4**	**T5**		**Treatment**	**Linear**	**Quadratic**
pH	6.93^a^	6.92^a^	6.96^a^	6.74^b^	6.75^b^	6.77^b^	0.014	< 0.001	< 0.001	< 0.001
NH_3_-N (mg/dL)	8.39	8.43	8.46	8.34	8.03	7.97	0.156	0.154	0.013	0.021
MCP (mg/mL)	0.38	0.42	0.42	0.46	0.40	0.43	0.037	0.685	0.367	0.381
**VFA concentration, mmol/L**										
Propionate	6.09^b^	7.94^ab^	8.35^a^	8.49^a^	6.24^b^	6.82^ab^	0.587	0.026	0.829	0.027
Butyrate	4.82^b^	5.44^ab^	5.30^ab^	6.20^a^	4.30^b^	4.40^b^	0.404	0.033	0.266	0.045
Acetate	22.38	27.17	26.70	31.25	22.17	22.75	2.764	0.182	0.737	0.116
Valerate	0.96	1.11	1.22	0.93	0.95	0.96	0.247	0.948	0.693	0.818
Ratio of acetate to propionate	3.68	3.46	3.25	3.63	3.54	3.34	0.292	0.890	0.644	0.881
TVFA	34.65	42.47	42.20	48.14	34.08	35.35	3.614	0.072	0.660	0.055
**VFA molar proportion (mol/100 mol)**										
Propionate	17.81	18.72	19.87	17.80	18.58	19.62	1.238	0.773	0.513	0.809
Butyrate	13.91	12.87	12.56	13.15	12.55	12.51	0.584	0.524	0.128	0.244
Acetate	64.32	63.80	63.18	64.45	64.78	63.85	1.585	0.984	0.879	0.981
Valerate	2.89	2.69	2.93	2.24	2.96	2.92	0.828	0.988	0.963	0.929

NH_3_-N, ammonia nitrogen; MCP, microbial protein; VFA, volatile fatty acids; TVFA, total volatile fatty acids.

CON: control group (no mixed isoacids added); T1:treatment 1 (0.1% mixed isoacids added); T2:treatment 2 (add 0.2% mixed isoacids added); T3:treatment 3 (add 0.3% mixed isoacids added); T4:treatment 4 (add 0.4% mixed isoacids added); and T5:treatment 5 (add 0.5% mixed isoacids added).

Different superscripts showed significant differences (*P* < 0.05).

^1^An explanation of abbreviations for some items in the table, such as MCP, NH_3_-N and VFA. ^2^An explanation of abbreviations for the groups, such as CON and 0.3% MI. ^3^An explanation that about *P*-value for significant differences.

### 3.2. Supplementation with mixed isoacids improved the growth performance and apparent digestibility of nutrients without affecting plasma biochemical and hormonal parameters in yaks

As shown in Table 4, supplementation with 0.3% MI significantly increased the ADG and ADMI when compared with the control (*P* < 0.05), but did not affect the FCR (*P* > 0.05). Yaks exhibited greater apparent digestibility of NDF and ADF in response to supplementation with 0.3% MI (*P* < 0.05). There was no significant difference in the apparent digestibility of EE, CP, Ca, and P between the treatments (*P* > 0.05). Meanwhile, supplementation with 0.3% MI had no effect on the concentration of plasma biochemical parameters and hormones (Supplementary Table 1), suggesting the nutrition metabolism of yaks themselves may not attribute to the growth-promoting effect of MI.

**Table 4:** Effects of mixed isoacids supplementation on the growth performance and apparent digestibility of nutrients in yaks.

**Items^1^**	**Groups** ^**2**^		**SEM**	***P*-value^2^**
	**CON**	**0.3% MI**		
**Growth performance**				
IBW (kg)	207.07	194.14	8.372	0.296
FBW (kg)	277.64	284.29	12.940	0.725
ADMI (kg/d)	6.10^b^	6.72^a^	0.170	0.012
ADG (kg)	0.83^b^	1.06^a^	0.066	0.037
FCR	7.76	6.41	0.482	0.110
**Apparent digestibility of nutrient %**				
NDF	70.86^b^	78.22^a^	1.938	0.024
ADF	62.03^b^	71.59^a^	2.744	0.042
CP	80.73	82.36	1.429	0.439
EE	88.92	88.93	1.354	0.995
Ca	49.38	52.22	3.952	0.619
P	76.22	71.20	2.779	0.293

IBW, initial body weight; FBW, final body weight; ADMI, average dry matter intake; ADG, average daily gain; FCR, feed conversion rate; NDF, neutral detergent fiber; ADF, acid detergent fiber; CP, crude protein; EE, ether extract; Ca, calcium; P, phosphorus.

CON, the basal diet; 0.3% MI, 0.3% mixed isoacids added to the basal diet on a dry matter basis.

Different superscribed letters showed significant differences (*P* < 0.05).

^1^An explanation of abbreviations for some items in the table, such as IBW, FBW, ADMI, ADG, FCR, NDF, ADF, CP, EE Ca and P. ^2^An explanation of abbreviations for the groups, such as CON and 0.3% MI. ^3^An explanation that about *P*-value for significant differences.

### 3.3. Supplementation with mixed isoacids tended to influence the ruminal fermentation characteristics in yaks

The concentrations of NH_3_-N, MCP, and VFAs in the rumen fluid of yaks are shown in Table 5. No significant difference was observed in NH_3_-N, MCP, and VFAs between the two groups (*P* > 0.05). Among the VFAs, the concentrations of acetate, propionate, and butyrate tended to decrease in response to the supplementation with 0.3% MI (0.05 < *P* < 0.1).

**Table 5:** Effects of mixed isoacids supplementation on ruminal fermentation characteristics in yaks.

**Items^1^**	**Groups** ^**2**^		**SEM**	***P*-value^3^**
	**CON**	**0.3% MI**		
NH_3_-N, mg/dL	13.13	14.39	1.016	0.423
MCP, mg/mL	0.85	0.85	0.079	0.979
**VFA concentration, mmol/L**				
Acetate	31.94	25.37	1.979	0.072
Propionate	6.96	5.51	0.529	0.079
Butyrate	3.89	3.03	0.280	0.056
Valerate	0.27	0.27	0.052	0.961
Isovalerate	1.37	1.41	0.293	0.824
Isobutyrate	1.04	1.07	0.171	0.765
Ratio of acetate to propionate	4.58	4.74	0.563	0.646
TVFA	45.89	36.67	7.183	0.064
**VFA proportion, mol/100 mol**				
Propionate	15.31	14.88	1.334	0.577
Butyrate	8.63	8.22	1.091	0.513
Acetate	69.90	69.37	2.680	0.716
Isovalerate	3.16	3.86	0.993	0.211
Valerate	0.63	0.74	0.163	0.221
Isovalerate	2.38	2.94	0.559	0.084

NH_3_-N, ammonia nitrogen; MCP, microbial protein; VFA, volatile fatty acids; TVFA, total volatile fatty acids.

CON, the basal diet; 0.3% MI, 0.3% mixed isoacids added to the basal diet on a dry matter basis.

^1^An explanation of abbreviations for some items in the table, such as MCP, NH_3_-N and VFA. ^2^An explanation of abbreviations for the groups, such as CON and 0.3% MI. ^3^An explanation that about *P*-value for significant differences.

### 3.4. Supplementation with mixed isoacids stimulated the formation of distinct ruminal bacterial communities in yaks

Illumina Miseq 16S rRNA gene sequencing obtained 48,307 ± 2,147 high-quality trimmed sequences per sample from 14 rumen fluid samples, which belonged to 2,612 OTUs, with an average sequence length of 418 bp (Supplementary Table 2). The information on barcodes from 14 rumen fluid samples is provided in Supplementary Table 2. A total of 1,937 OTUs (74.16%) were commonly shared by the control and 0.3% MI groups, while 276 OTUs (10.57%) were specific to the control group and 399 OTUs (15.28%) were specific to the 0.3% MI group (Supplementary Figure 2). The composition of the ruminal bacterial community was visualized using the Circos plot (Supplementary Figure 3). Both groups displayed similar bacterial composition at the phylum level (Supplementary Figure 3A), family level (Supplementary Figure 3B), and genus level (Supplementary Figure 3C).

**Figure 1:**
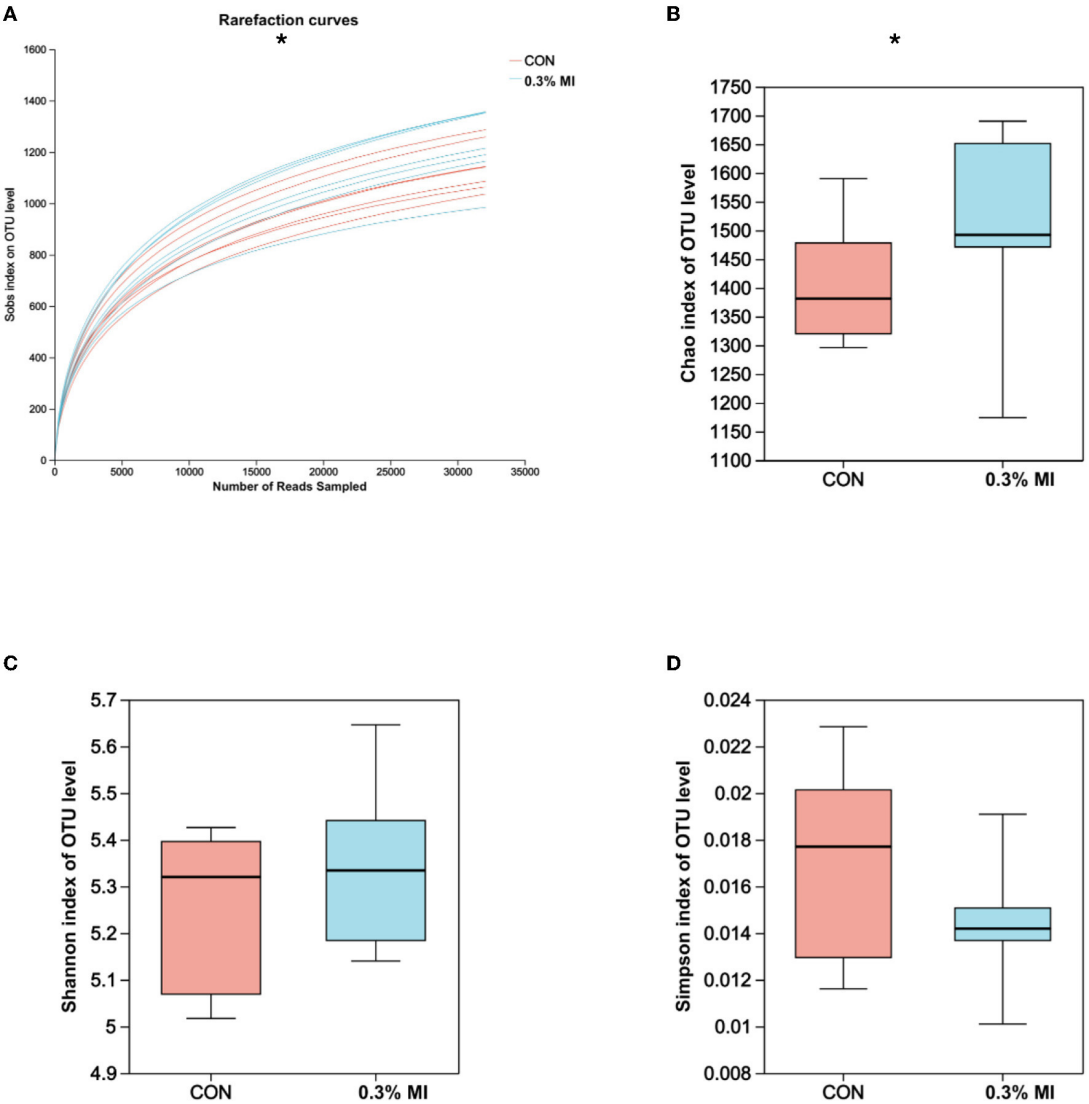
Effects of mixed isoacids supplementation on rumen bacterial richness and diversity in yaks. **(A)** Rarefaction curve based on Sobs indices; **(B)** Boxplot of Chao indices; **(C)** Boxplot of Shannon indices; **(D)** Boxplot of Simpson indices. CON, the basal diet; 0.3% MI, 0.3% mixed isoacids added to the basal diet on a dry matter basis. Significance was tested using the independent two-group Wilcoxon rank-sum tests. ^*^P < 0.05, *n* = 7 per group.

**Figure 2:**
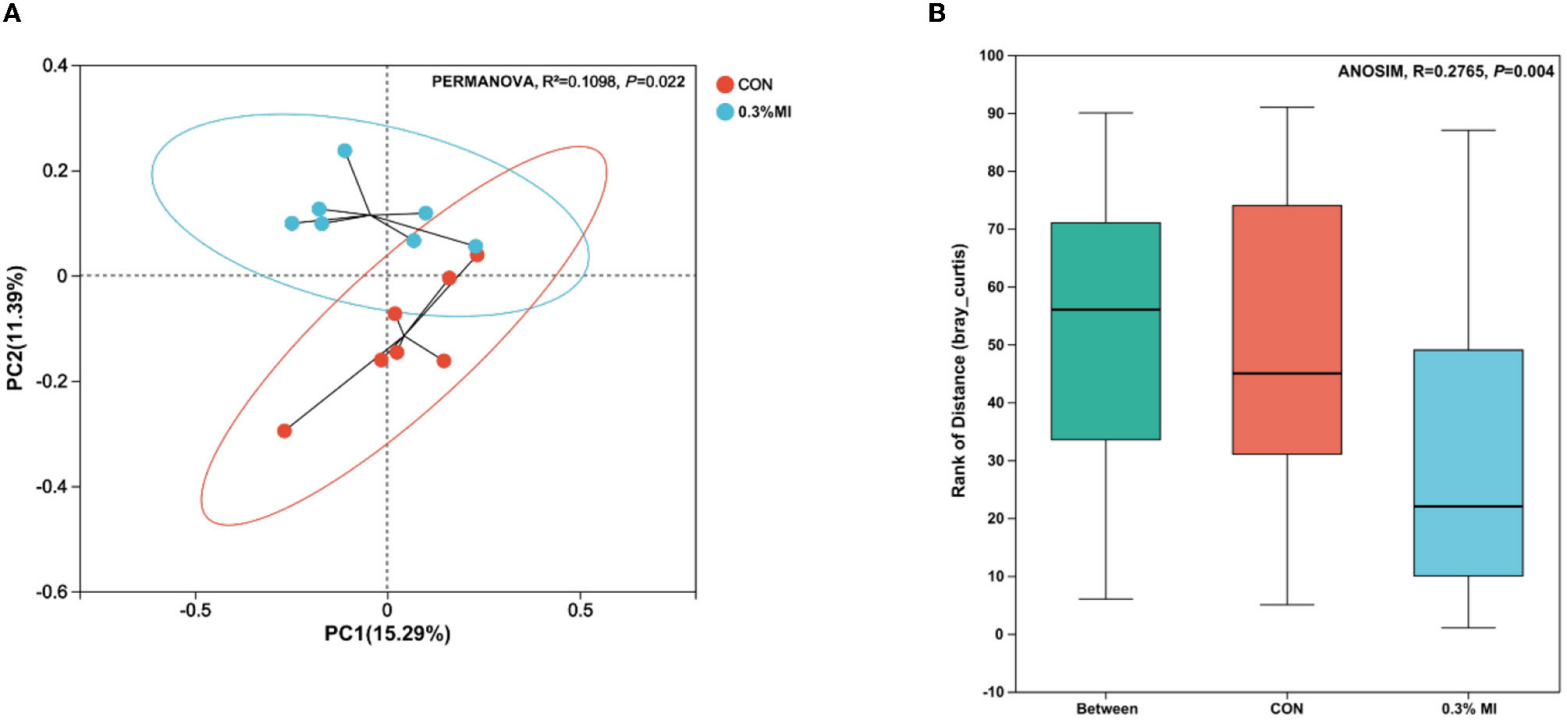
Effects of mixed isoacids supplementation on rumen bacterial community similarity. **(A)** Principal coordinates analysis (PCoA) plot based on the Bray-Curtis distance metric. Difference between clusters is measured by the permutational multivariate analysis of variance (PERMANOVA). **(B)** Distances box plot depicted the analysis of similarities (ANOSIM) between the CON group and the 0.3% MI group. CON, the basal diet; 0.3% MI, 0.3% mixed isoacids added to the basal diet on a dry matter basis.

The rarefaction curves based on Sobs indices reached the saturation stage with the increasing number of sequenced reads (Figure 1A), suggesting that the majority of rumen bacterial members were captured from rumen fluid samples in this experiment. However, the bacterial community richness (Chao index, Figure 1B) and diversity (Shannon and Simpson indices, Figures 1C, D) did not differ between the control and 0.3% MI groups (*P*>0.05). Principal coordinates analysis (PCoA) based on the Bray-Curtis distance metric showed that supplementation with 0.3% MI induced the rumen bacteria to form significantly different communities when compared with the control group (PERMANOVA, R^2^ = 0.1098, *P* = 0.022, Figure 2A). A similarities analysis showed that the difference in bacterial communities between the groups was greater than the difference within a group (ANOSIM, R^2^ = 0.2765, *P* = 0.004, Figure 2B).

**Figure 3:**
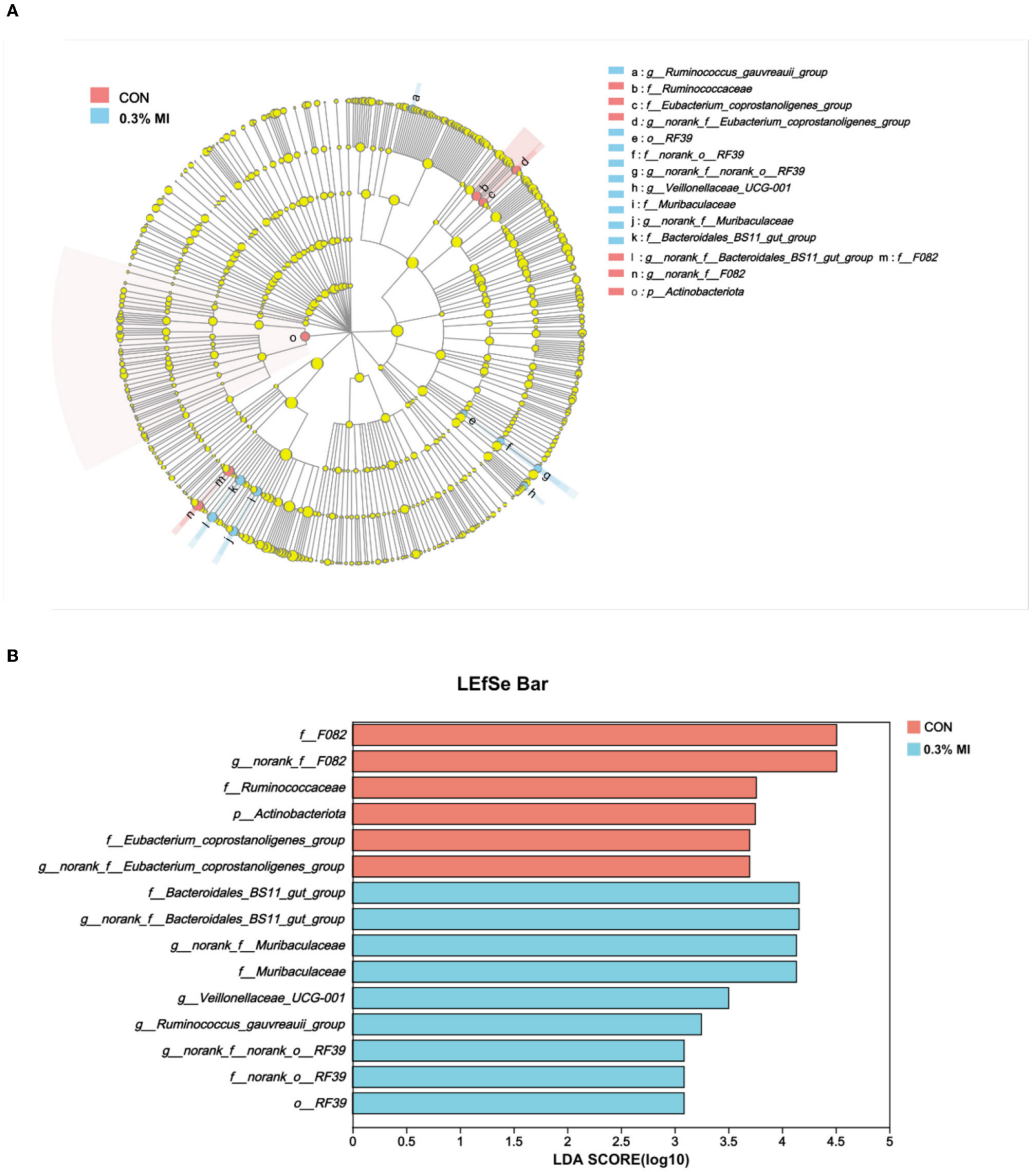
Effects of mixed isoacids supplementation on the most differentially abundant rumen bacterial taxa in yaks. **(A)** Cladogram plot of linear discriminant analysis (LDA) effect size (LEfSe) results from phylum to genus level. **(B)** Histogram of linear discriminant analysis (LDA) reveals the most differentially abundant taxa between the groups. CON, the basal diet; 0.3% MI, 0.3% mixed isoacids added to the basal diet on a dry matter basis.

The most discriminative taxa responding to supplementation with 0.3% MI was identified using the linear discriminant analysis (LDA) effect size (LEfSe). Fifteen differentially abundant taxa from phylum to genus level were discovered as high-dimensional biomarkers for separating rumen bacterial communities between the two groups (Figure 3A). Nine of these taxa were higher, and six were lower in the 0.3% MI group than in the CON group (Figure 3A). Dietary supplementation with 0.3% MI showed a significantly higher abundance of f__*Bacteroidales*_BS11_gut_group, g__norank_f__*Bacteroidales*_BS11_gut_group, g__norank_f__*Muribaculaceae*, f__*Muribaculaceae*, g__*Veillonellaceae*_UCG-001, g__*Ruminococcus_gauvreauii*_group, g__norank_f__norank_o__RF39, f__norank_o__RF39, and o__RF39 (*P* < 0.05). In the CON group group, f__F082, g__norank_f__F082, f__*Ruminococcaceae*, p__*Actinobacteriota*, f__*Eubacterium_coprostanoligenes*_group, and g__norank_f__*Eubacterium_coprostanoligenes*_group were significantly enriched, from the family to the genus level (Figures 3A, B, *P* < 0.05). Besides, a Student's *t*-test was performed to detect all differentially abundant taxa in the top 100 (Supplementary Table 3).

### 3.5. Apparent digestibility of NDF and ADF were positively associated with ruminal differentially abundant taxa

**Figure 4:**
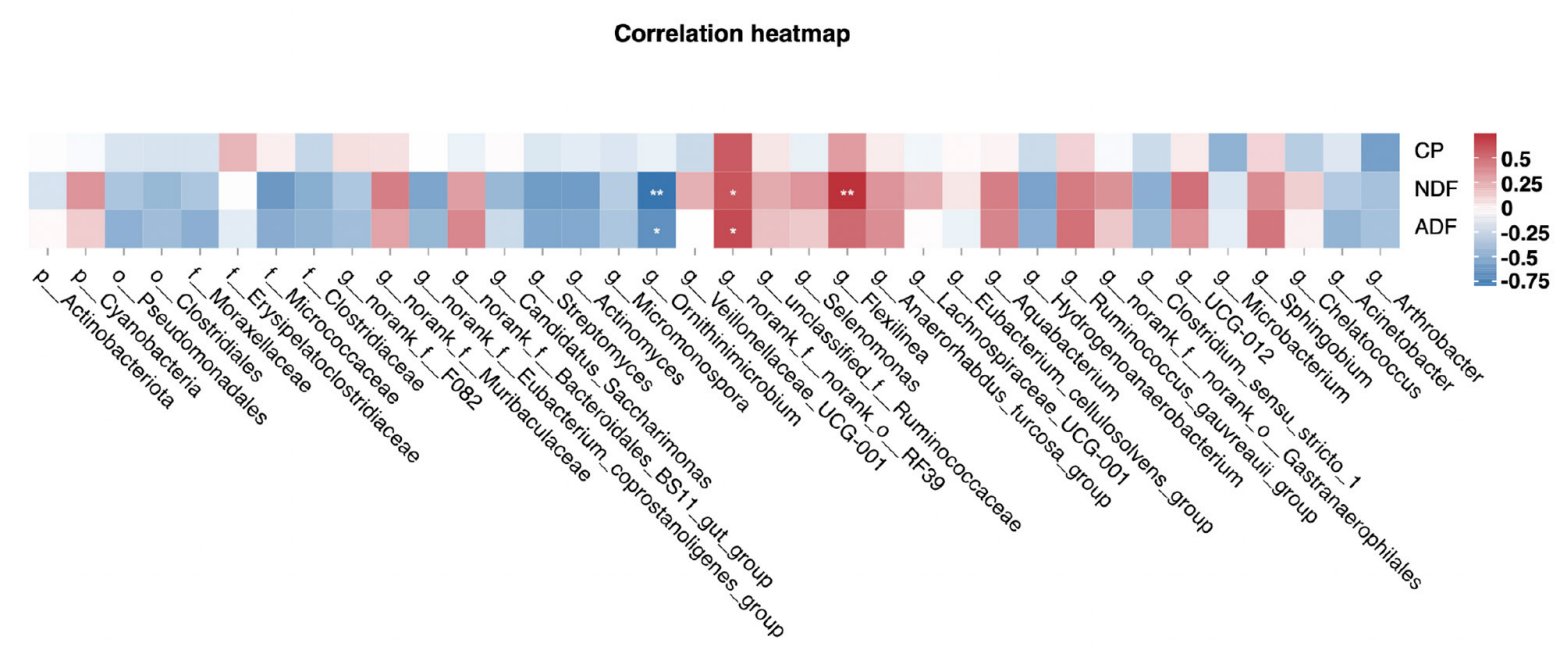
The correlations of feed digestibility with the bacterial community from phylum to genus level. The heatmap shows the absolute value of Spearman's correlation *R* > 0.1. CP, crude protein; NDF, neutral detergent fiber; ADF, acid detergent fiber. Asterisk indicates the significance of Spearman's correlation, *P < 0.05, **P < 0.01, *n* = 7 per group.

Differentially abundant taxa at different levels, identified by the Student's *t*-test, were selected for the correlation analysis with apparent digestibility of nutrients. Spearman's correlation indicated that g__*Flexilinea* and g__norank_f__norank_o__RF39 were significantly positively correlated with the NDF digestibility (*P* < 0.05); and g__norank_f__norank_o__RF39 was significantly positively correlated with the ADF digestibility (*P* < 0.05). In addition, g__*Ornithinimicrobium* was significantly negatively correlated with the ADF and NDF digestibility (*P* < 0.05, Figure 4).

## 4. Discussion

Improving the fiber utilization by yaks is important for highland yak farming in winter when forage is severely scarce (Dong et al., 2006). The rumen is well known as a natural bioreactor for its high efficiency of fiber degradation by rumen microbiota (Wang et al., 2020). Isoacids are a batch of amino acid-derived metabolites in the rumen that act as a nutrient factor for rumen fiber-degrading bacteria, thereby facilitating VFAs production and fiber utilization (Liu et al., 2009, 2014; Roman-Garcia et al., 2021a,c). Concentrations of butyrate and propionate, as well as the degradation of NDF and ADF, tended to increase and then decrease with increasing dose of MI in this study. These results are consistent with those of Wang et al. (2012), who obtained results from an *in vitro* fermentation assay supplemented with 2-methylbutyrate, suggesting that a higher dose of MI may dampen the *in vitro* fermentation characteristics. The supplementation level of 0.3% could be an optimal dose, as it achieved the highest propionate and butyrate content, as well as NDFD and ADFD, indicating that the effect of MI on rumen fermentation parameters in yaks is related to its supplementation level.

Supplementation with isoacids alone or in combination could improve feed fiber digestibility and average daily gain in beef cattle and dairy calves (Wang et al., 2012; Liu C. S. et al., 2016; Liu et al., 2018). Improved digestibility of NDF and NDF responsive to MI makes more fermentation metabolites, such as VFAs, available in the rumen. For example, Wang et al. (2018b) found that supplementation with 16.8 g of 2-methylbutyrate per steer per day increased the molar ratio of effectively degradable NDF and rumen acetate. Similar results were observed in this study, suggesting that supplementation with 0.3% MI could significantly improve the apparent digestibility of NDF and ADF, thereby increasing the ADG and ADMI in yaks. However, ruminal production of VFA was not affected by the addition of MI, which may be due to factors such as diet composition, rumen microbial species, pen temperature, and the amount or composition of MI (Wang et al., 2016a,b). NH_3_-N is a product of the fermentation and degradation of dietary proteins and is an essential component for microbial synthesis of MCP in the rumen (Garcia-Gonzalez et al., 2010; Thao et al., 2014; Lv et al., 2020). However, no difference in the response of NH_3_-N concentration to MI was observed in this study, which may be related to the amount of water consumed by the yaks or the amount or composition of the MI supplement.

After a long period of natural selection, the yak rumen microbiota has evolved a higher capacity for fiber degradation (Ma et al., 2020). Thus, the yak rumen may harbor a unique microbiome for efficient conversion of feed fiber. Isoacids improve the digestibility of feed fiber, mainly by increasing the activity of cellulase and cellulose-producing bacteria in the rumen. For example, Liu C. S. et al. (2016) reported that supplementation with 6.0 g of isovalerate per calf per day increased the activities of caboxymethylcellulase, cellobiase, xylanase and pectinase and the relative amounts of *Butyrivibrio fibrisolvens, Ruminococcus albus, Fibrobacter succinogenes*, and *Ruminococcus flavefaciens* in dairy calves at 90 days of age. Similarly, Wang et al. (2019) found that supplementation with 6 g of isobutyrate per calf per day increased the activities of cellobiase, xylanase, pectinase, b-amylase protease, and CMCase in post-weaned calves and the population of *B. fibrisolvens* in pre- and post-weaned calves. Rather than looking at changes in the rumen microbiota as a whole, these studies have focused on changes in the abundance of specific microbes. In this study, beta-diversity by PCoA showed that supplementation with 0.3% MI induced the rumen bacteria to form significantly different communities, indicating that 0.3% MI significantly altered the rumen microbial structure, which may be related to the fact that the microbial 16S rRNA gene was less diverse in the yak rumen than that in the bovine rumen (An et al., 2005). This resulted in a significant effect of 0.3% MI on some uncultured fiber-degrading bacteria in the yak rumen and ultimately caused a shift in the structure of the rumen microbiota.

The LEfSe analysis showed that some biomarker taxa such as norank_f__*Bacteroidales_*BS11_gut_group, norank_f__*Muribaculaceae, Veillonella- ceae_*UCG-001, and *Ruminococcus_gauvreauii*_group were found in 0.3% of the MI group. The *Bacteroidales* belong to the phyla of *Bacteroidetes*, which act mainly on steroids, polysaccharides, and bile acids, which contribute to the absorption of polysaccharides and proteins by the body (Backhed et al., 2005). Therefore, we can speculate that the norank_f__*Bacteroidales_*BS11_gut_group may be a fiber-degrading bacterium. One study found that norank_f__*Muribaculaceae* was positively correlated with milk yield (Sun et al., 2019), and some studies found that isoacids can increase milk production in dairy cows (Otterby et al., 1990). The higher relative expression of norank_f__*Muribaculaceae* in the 0.3% MI group suggests that 0.3% MI may increase milk production in yaks, but no studies have been reported on this aspect. In addition, *Muribaculaceae* can also degrade carbohydrates (He et al., 2022), which corresponds to a higher apparent digestibility of NDF and ADF in the 0.3% MI group than in the control group, suggesting that 0.3% MI can promote the growth of rumen fibrinolytic bacteria in yaks, which is consistent with other studies (Moharrery and Das, 2001; Firkins, 2010; Liu et al., 2014; Wang et al., 2015). *Veillonellaceae_*UCG-001 belong to the phylum *Firmicutes*, which contains many fibrolytic bacteria (Chen et al., 2022). According to a remarkably higher apparent digestibility of NDF and ADF in the 0.3% MI group than that in the control group. It is speculated that *Veillonellaceae_*UCG-001 may also be a type of fibrolytic bacteria in the yak rumen. *Ruminococcus* also belongs to the phylum *Firmicutes*, which can use cellulose and hemicellulose as substrates to produce VFA (Liu et al., 2019). The *in vitro* tests in this study showed a significant increase in propionate acid and butyrate in the 0.3% MI group and a trend toward higher TVFA, which may be related to the increased relative abundance of the *Ruminococcus_gauvreauii_group*. In addition, supplementation with 0.3% MI reduced the relative abundance of norank_f__F082 by 49.98%, which was reported to be negatively correlated with feed fiber concentration and digestibility in yaks (Yi et al., 2022), suggesting that supplementation with MI may increase the available fiber content and digestibility in the rumen of yaks.

The correlation analysis showed that g__*Flexilinea* and g__norank_f__norank_o__RF39 were positively correlated with the apparent digestibility of NDF. g__*Flexilinea* was reported as a filamentous strictly anaerobic Gram-negative bacterium which could digest all kinds of carbohydrates (Wang et al., 2021). In addition, one study has reported that the abundance of g__norank_f__norank_o__RF39 was increased with the increase of NDF digestibility (Liu et al., 2023). We speculate that supplementation with 0.3% MI significantly improved the apparent digestibility of feed fiber, which was associated with the change in the abundance of g__*Flexilinea* and g__norank_f__norank_o__RF39.

## 5. Conclusion

The optimum dose of MI was found to be 0.3% based on the *in vitro* experiment, which significantly reduced ruminal pH, increased NDFD, ADFD, propionate, and butyrate concentrations, and tended to increase the content of TVFA. Supplementation with 0.3% MI significantly improved the apparent digestibility of feed fiber and altered the ruminal bacterial diversity, which was positively correlated with the abundance of g__*Flexilinea* and g__norank_f__norank_o__RF39.

## Data availability statement

The datasets presented in this study can be found in online repositories. The names of the repository/repositories and accession number(s) can be found below: NCBI - Genome Sequence Archive (GSA) database, and the BioProject accession number is PRJCA016021.

## Ethics statement

The animal study was reviewed and approved by Animal Care and Use Committee of Southwest Minzu University.

## Author contributions

YG and ZP contributed to conception and design of the study. FJ and XM performed the *in vitro* fermentation experiment. FJ, YY, ZH, YL, and AH conducted the animal experiment. YG and FJ performed the statistical analysis and wrote the manuscript. All authors contributed to manuscript revision, read, and approved the submitted version.
