# Supplementary material for: Isoacids supplementation improves growth performance and feed fiber digestibility associated with ruminal bacterial community in yaks
Source: Front Microbiol. 2023 Jun 15;14:1175880. doi: 10.3389/fmicb.2023.1175880 (PMC10311502; doi:10.3389/fmicb.2023.1175880)
Supplement: Supplementary file 1 [file Data_Sheet_1.pdf]

Supplementary Data

Table S1. Effects of mixed isoacids supplementation on the concentrations of plasma biochemical and hormonal parameters in yaks

Items[1]	Groups[2]		SEM	*P*-value[3]
	CON	0.3% MI		
TP (g/L)	89.60	92.01	8.830	0.851
ALB (g/L)	39.66	40.50	2.490	0.819
GLO (g/L)	49.94	51.51	6.480	0.867
ALT (U/L)	38.37	44.06	5.875	0.511
AST (U/L)	101.11	93.78	10.643	0.682
GLU (mmol/L)	5.21	6.24	0.666	0.316
TC (mmol/L)	2.65	2.71	0.327	0.903
TG (mmol/L)	0.38	0.45	0.056	0.463
UREA (mmol/L)	7.38	8.28	0.665	0.363
EGF (pg/mL)	577.15	569.51	42.275	0.901
TGF-β (pg/mL)	210.96	210.49	15.600	0.983
β-HB (μmol/L)	17.59	17.39	1.069	0.898
FFA (μmol/L)	235.91	285.25	18.658	0.089
IGF-1 (ng/mL)	171.77	195.84	16.657	0.339
INS (mIU/L)	17.01	17.64	0.698	0.547
GH (ng/mL)	13.60	13.06	0.503	0.500

1) TP, total protein; ALB, albumin protein; GLO, globulin; ALT, alanine aminotransferase; AST, aspartate aminotransferase; GLU, glucose; TC, total cholesterol; TG, triglyceride; EGF, epidermal growth factor; TGF-β, transforming growth factor-beta; β-HB, beta-hydroxybutyric acid, FFA, free fatty acid; IGF-1, insulin-like growth factor 1; INS, insulin; GH, growth hormone.
2) CON, the basal diet; 0.3% MI, 0.3% mixed isoacids added to the basal diet on a dry matter basis.
3) Different superscribed letters showed significant differences ($P<0.05$)

Table S2. Barcode, number and length of sequences obtained by Illumina Miseq 16S rRNA gene sequencing from rumen fluid samples in yaks in response to mixed isoacids supplementation

Sample ID	Barcode	Sequence_number	Base_number	Mean_length	Min_length	Max_length
D1	ZB38	49880	20818500	417.3717	252	445
D2	ZB39	57540	24152842	419.7574	219	513
D3	ZB40	49071	20499915	417.7603	230	452
D4	ZB41	38624	16145657	418.0214	219	431
D5	ZB42	45502	19009787	417.7792	225	526
D6	ZB43	34801	14460158	415.5098	219	431
D7	ZB44	51204	21379146	417.5288	226	505
Y1	ZB45	38586	16010392	414.9275	212	482
Y2	ZB46	45304	19008891	419.5853	333	437
Y3	ZB47	41474	17399700	419.5327	227	495
Y4	ZB48	54474	22803877	418.6195	252	478
Y5	ZB1	62571	26115637	417.3761	244	463
Y6	ZB2	55839	23306881	417.3943	252	431
Y7	ZB3	51423	21599335	420.0326	201	460

Table S3. Effects of mixed isoacids supplementation on the differentially abundant rumen bacterial taxa in yaks (Student's t-test)

Items	Groups[1]		SEM	*P*-value[2]
	CON	0.3% MI		
Phylum level				
p__*Actinobacteriota*	2.211[a]	1.251[b]	0.422	0.042
p__*Cyanobacteria*	0.029[a]	0.070[b]	0.018	0.036
Class level				
c__*Saccharimonadia*	0.646[a]	0.460[b]	0.069	0.020
c__*Vampirivibrionia*	0.027[a]	0.068[b]	0.017	0.035
c__*Anaerolineae*	0.033[a]	0.055[b]	0.008	0.017
Order level				
o__RF39	0.458[b]	0.656[a]	0.067	0.012
o__*Saccharimonadales*	0.646[a]	0.460[b]	0.069	0.020
o__*Pseudomonadales*	0.191[a]	0.062[b]	0.058	0.045
o__*Gastranaerophilales*	0.027[b]	0.068[a]	0.017	0.035
o__*Anaerolineales*	0.033[b]	0.054[a]	0.008	0.018
o__*Streptomycetales*	0.042[a]	0.014[b]	0.012	0.042
o__*Clostridiales*	0.033[a]	0.012[b]	0.008	0.023
o__*Micromonosporales*	0.003[a]	0.000[b]	0.001	0.027
Family level				
f__*Bacteroidales*_BS11_gut_group	1.254[b]	4.269[a]	1.375	0.049
f__F082	12.086[a]	5.265[b]	1.893	0.004
f__*Muribaculaceae*	1.505[b]	4.022[a]	0.969	0.023
f__*Moraxellaceae*	0.187[a]	0.059[b]	0.056	0.043
f__*Eubacterium_coprostanoligenes*_group	2.297[a]	1.283[b]	0.258	0.002
f__*Erysipelatoclostridiaceae*	0.762[a]	0.359[b]	0.174	0.039
f__*Saccharimonadaceae*	0.646[a]	0.460[b]	0.069	0.020
f__*Micrococcaceae*	0.122[a]	0.026[b]	0.041	0.039
f__*Streptomycetaceae*	0.042[a]	0.014[b]	0.012	0.042
f__*Clostridiaceae*	0.033[a]	0.010[b]	0.008	0.017
f__*Micromonosporaceae*	0.003[a]	0.000[b]	0.001	0.027
f__norank_o__RF39	0.458[b]	0.656[a]	0.067	0.012
f__norank_o__*Gastranaerophilales*	0.027[b]	0.068[a]	0.017	0.035
f__*Anaerolineaceae*	0.033[b]	0.054[a]	0.008	0.018
Genus level				
g__norank_f__F082	12.086[a]	5.265[b]	1.892	0.004
g__norank_f__*Muribaculaceae*	1.504[b]	4.022[a]	0.969	0.023
g__norank_f__*Eubacterium_coprostanoligenes*_group	2.297[a]	1.283[b]	0.258	0.002
g__norank_f__*Bacteroidales*_BS11_gut_group	1.254[b]	4.269[a]	1.375	0.049
g__*Candidatus_Saccharimonas*	0.646[a]	0.460[b]	0.069	0.020
g__*Streptomyces*	0.042[a]	0.014[b]	0.012	0.042
g__*Actinomyces*	0.003[a]	0.000[b]	0.001	0.033
g__*Micromonospora*	0.003[a]	0.000[b]	0.001	0.027
g__*Ornithinimicrobium*	0.002[a]	0.000[b]	0.001	0.028
g__*Veillonellaceae*_UCG-001	0.560[b]	1.226[a]	0.221	0.011
g__norank_f__norank_o__RF39	0.458[b]	0.656[a]	0.067	0.012
g__unclassified_f__*Ruminococcaceae*	0.069[b]	0.158[a]	0.033	0.018
g__*Selenomonas*	0.058[b]	0.115[a]	0.024	0.034

g__*Flexilinea*	0.033^{b}	0.054^{a}	0.008	0.018
g__*Anaerorhabdus_furcosa*_group	0.020^{b}	0.047^{a}	0.011	0.039
g__*Lachnospiraceae*_UCG-001	0.000^{b}	0.011^{a}	0.005	0.046
g__*Eubacterium_cellulosolvens*_group	0.000^{b}	0.010^{a}	0.004	0.027
g__*Aquabacterium*	0.000^{b}	0.007^{a}	0.002	0.025
g__*Hydrogenoanaerobacterium*	0.002^{a}	0.000^{b}	0.001	0.028
g__*Ruminococcus_gauvreauii*_group	0.380^{b}	0.763^{a}	0.163	0.037
g__norank_f__norank_o__*Gastranaerophilales*	0.027^{b}	0.068^{a}	0.017	0.035
g__*Clostridium_sensu_stricto*_1	0.030^{a}	0.009^{b}	0.008	0.022
g__UCG-012	0.006^{b}	0.016^{a}	0.004	0.034
g__*Microbacterium*	0.015^{a}	0.004^{b}	0.005	0.046
g__*Sphingobium*	0.000^{b}	0.003^{a}	0.001	0.033
g__*Chelatococcus*	0.000^{b}	0.003^{a}	0.001	0.033
g__*Acinetobacter*	0.167^{a}	0.042^{b}	0.052	0.034
g__*Arthrobacter*	0.027^{a}	0.011^{b}	0.008	0.049

1) CON, the basal diet; 0.3% MI, 0.3% mixed isoacids added to the basal diet on a dry matter basis.

2) Different superscribed letters showed significant differences (P<0.05)

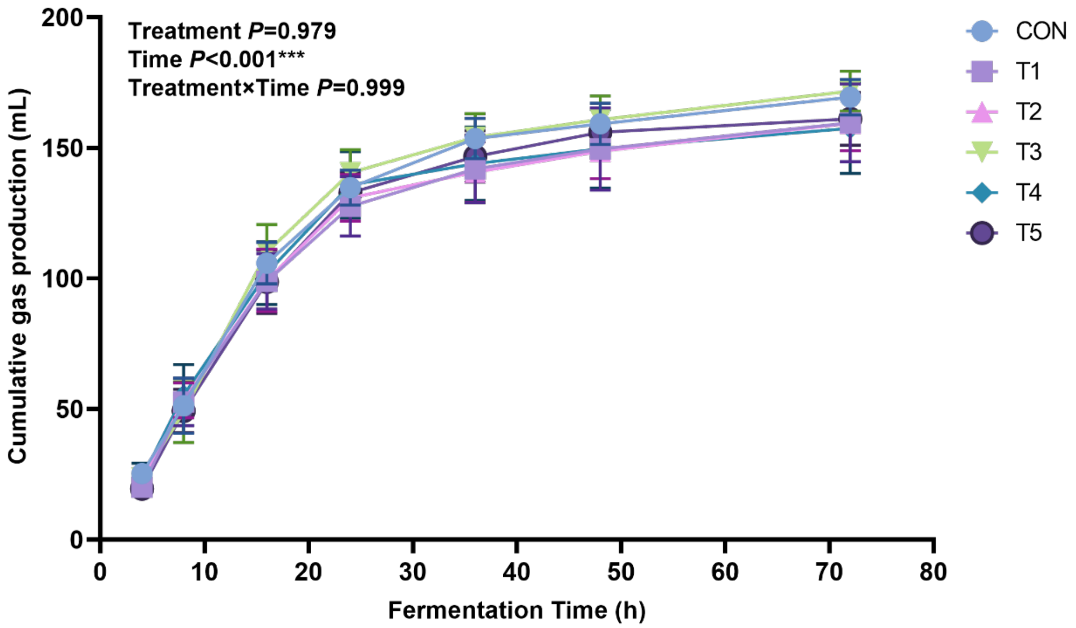

Figure S1. Cumulative gas production of *in vitro* fermentation experiment. CON: control group (no mixed isoacids added); T1, treatment 1 (0.1% mixed isoacids added); T2, treatment 2 (0.2% mixed isoacids added); T3, treatment 3 (0.3% mixed isoacids added); T4, treatment 4 (0.4% mixed isoacids added); T5, treatment 5 (0.5% mixed isoacids added).

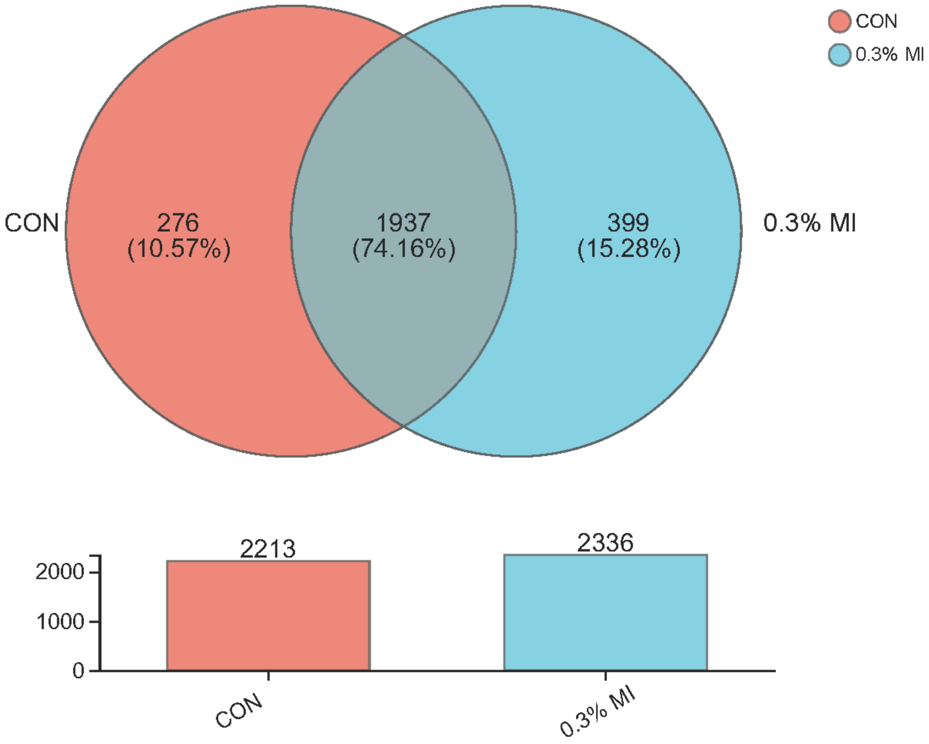

Figure S2. The shared and specific operational taxonomic units (OTUs) in the control and mixed isoacids supplementation group are depicted by the Venn diagram. CON, the basal diet; 0.3% MI, 0.3% mixed isoacids added to the basal diet on a dry matter basis.

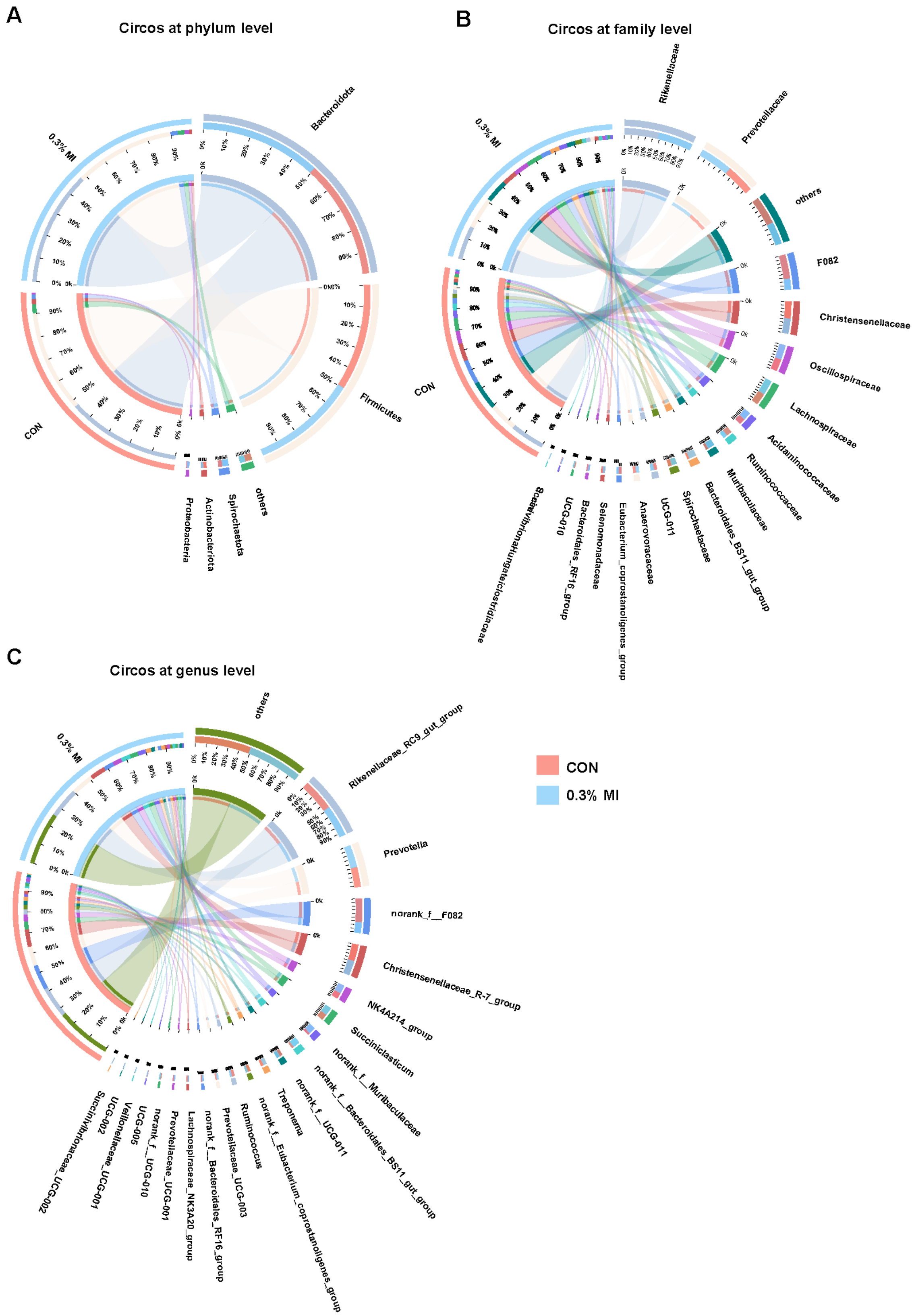

Figure S3. Effects of mixed isoacids supplementation on the composition of the bacterial community in yaks. Sample-to-genera relationship was visualized by the Circos plot. (A) Bacterial composition at the phylum level. (B) Bacterial composition

at the family level. (C) Bacterial composition at the genus level. CON, the basal diet; 0.3% MI, 0.3% mixed isoacids added to the basal diet on a dry matter basis.
